# Air contamination of households versus hospital inpatient rooms occupied by severe acute respiratory coronavirus virus 2 (SARS-CoV-2)–positive patients

**DOI:** 10.1017/ice.2021.45

**Published:** 2021-02-04

**Authors:** L. Silvia Munoz-Price, Frida Rivera, Nathan Ledeboer

**Affiliations:** 1 Division of Infectious Diseases, Department of Medicine, Medical College of Wisconsin, Milwaukee, Wisconsin; 2 Department of Pathology, Medical College of Wisconsin, Milwaukee, Wisconsin

## Abstract

The household setting has some of the highest coronavirus disease 2019 (COVID-19) secondary-attack rates. We compared the air contamination in hospital rooms versus households of COVID-19 patients. Inpatient air samples were only positive at 0.3 m from patients. Household air samples were positive even without a COVID-19 patient in the proximity to the air sampler.

Severe acute respiratory coronavirus virus 2 (SARS-CoV-2) is transmitted primarily by respiratory droplets and contact with contaminated surfaces and fomites.^1^ Airborne transmission is still a controversial topic among the scientific community. A few studies have successfully identified SARS-CoV-2 in the air of hospital rooms using real-time reverse-transcriptase polymerase chain reaction (RT-PCR) or viral cultures.^2-5^ However, air contamination in households has yet to be characterized. The household setting has high secondary-attack rates, and members of the same household have been shown to experience up to 10 times greater risk of coronavirus disease 2019 (COVID-19) than other contacts (ie, healthcare workers, workplace contacts, and nonhousehold contacts).^6-8^

Understanding the degree of air contamination in household settings would help us tailor prevention interventions in these high-risk settings. To address this knowledge gap, our study aimed to characterize and compare the presence of SARS-CoV-2 in air samples obtained in household settings against air samples obtained in inpatient rooms both selected based on the presence of SARS-CoV-2–positive patients.

## Methods

This study was performed at Froedtert Memorial Lutheran Hospital, a 607-bed academic medical center affiliated with the Medical College of Wisconsin. This inpatient facility has 6 intensive care units (ICUs) with 150 ICU beds. During the pandemic, a few units were designated for cohorting SARS-CoV-2–positive patients, including the medical ICU, the cardiovascular ICU, and a couple of general medical-surgical units. All of these units were set for at least 6 air changes per hour and for negative pressure relative to the hallways.

A convenience sample of rooms was selected based on the presence of patients with positive SARS-CoV-2 RT-PCR tests. Households were identified based on the presence of at least 1 household member with symptoms compatible with COVID-19 and at least one positive SARS-CoV-2 RT-PCR test. All study participants had a positive SARS-CoV-2 test in the 7 days preceding air sampling.

### Air sampling and molecular testing

Air samples were collected using the Sartorius MD8 airscan sampling device (SartoriusAG, Göttingen, Germany) with sterile gelatin filters (80 mm in diameter and 3 μm pore size (SartoriusAG). Briefly, the air sampler was positioned 0.305–1.83 m from the patient’s head to collect 1,000–4,000 L (50 L/minute). We evaluated shorter distances and higher volumes until we were able to detect SARS-CoV-2 in air samples. For samples that obtained 4,000 L, 2 air samplers were used concomitantly for 40 minutes (50 L/minute; 2,000 L each). Gelatin filters were placed in 6 mL viral transport media (VTM) (Remel M4RT, ThermoFisher, Lenexa, KS). If 2 air samplers were used concomitantly to achieve 4,000 L, then both gelatin membranes were placed in a single container with 6 mL VTM. These plates were incubated at 37^o^C for 1 minute to allow the gelatin filter to dissolve. This mixture was then spun in a vortex machine and centrifuged at 13,000×*g* for 1 minute, and 1 mL supernatant was used for nucleic acid extraction. Nucleic acid extraction and RT-PCR were performed on the Cobas 6800 (Roche, Indianapolis, IN) according to the manufacturer’s emergency use authorization (EUA)–approved product insert. For patient specimens, combined nasopharyngeal and oropharyngeal swabs were collected from each patient, and both the nasopharyngeal and oropharyngeal swabs were placed into 3 mL VTM (Copan, Murrieta, CA). RT-PCR was performed to detect the presence of SARS-CoV-2 on each sample using the Cobas 6800 according to the manufacturer’s EUA-approved product insert.

## Results

We included 25 air samples from 15 inpatient rooms (16 samples) and 5 households (9 samples) where SARS-CoV-2–positive patients were housed (Table 1). Of 16 air samples from inpatient rooms, 2 (12.5%; 2 inpatient rooms) were SARS-CoV-2 positive, and of 9 household samples, 5 (55.5%; 3 households) had SARS-CoV-2 detected (odds ratio, 8.75; 95% confidence interval, 1.21–63.43; *P* = .058). All samples had cycle threshold levels >30.

**Table 1.** Presence of SARS-CoV-2 in Households and Inpatient Hospital Rooms Occupied by SARS-CoV-2 Positive Patients

Patient/ Household	Unit/ Room	Windows Opened Prior to Testing	Air Conditioning or Fans On	COVID-19 Symptoms on Day of Testing	Date of Symptoms Onset	Date of Admission	Oxygen Requirement	Last SARS-CoV-2 Positive Test	Date of Sampling	Sampling Time, min	Air Volume Sampled, L	Distance from Patient, m	Size of Room, m^3^	RT-PCR Result of Air Sample	CT values for air samples
1	COVID unit (general medicine)	NA	NA	No COVID-19 symptoms	NA	8/16/20	No	8/16/20	8/19/20	20	1,000	1.83	260.1	Negative	NA
2	COVID unit (general medicine)	NA	NA	Pneumonia	8/13/20	8/16/20	High flow nasal cannula	8/16/20	8/19/20	20	1,000	1.83	260.1	Negative	NA
3	COVID unit (general medicine)	NA	NA	No COVID-19 symptoms	NA	8/14/20	No	8/15/20	8/19/20	20	1,000	1.83	260.1	Negative	NA
4	COVID unit (ICU)	NA	NA	Hypoxic respiratory failure	8/18/20	8/19/20	BiPAP	8/19/20	8/26/20	20	1,000	0.91	89.2	Negative	NA
5	COVID unit (ICU)	NA	NA	Hypoxic respiratory failure	8/10/20	8/16/20	High flow nasal cannula	8/14/20	8/26/20	20	1,000	0.91	89.2	Negative	NA
6	Non-COVID unit (ICU)	NA	NA	Septic shock unclear etiology	Unknown	8/21/20	Intubated	8/21/20	8/26/20	20	1,000	0.91	157	Negative	NA
7	COVID unit (general medicine)	NA	NA	Hypoxic respiratory failure	Unknown	9/2/20	Nasal cannula	9/2/20	9/4/20	20	1,000	0.91	260.1	Negative	NA
7	COVID unit (general medicine)	NA	NA	Hypoxic respiratory failure	Unknown	9/2/20	Nasal Cannula	9/2/20	9/4/20	40	2,000	0.91	260.1	Negative	NA
8	COVID unit (general medicine)	NA	NA	Hypoxic respiratory failure	9/4/20	8/25/20	No	9/8/20	9/9/20	40	2,000	0.91	260.1	Negative	NA
9	COVID unit (general medicine)	NA	NA	Hypoxic respiratory failure	9/7/20	9/8/20	High flow nasal cannula	9/8/20	9/9/20	40	2,000	0.91	260.1	Negative	NA
10	COVID unit (general medicine)	NA	NA	Pneumonia	9/5/20	9/1/20	Nasal Cannula	9/8/20	9/9/20	40	2,000	0.91	260.1	Negative	NA
11	COVID unit (ICU)	NA	NA	Pneumonia	9/7/20	9/6/20	Intubated	9/8/20	9/9/20	40	2,000	0.91	89.2	Negative	NA
12	COVID unit (ICU)	NA	NA	Cough, fatigue, fever, diarrhea	9/12/20	9/21/20	Nasal Cannula	9/22/20	9/22/20	40	4,000	0.91	89.2	Negative	NA
13	COVID unit (ICU)	NA	NA	Pneumonia	Unknown	9/21/20	Nasal Cannula	9/21/20	9/22/20	40	4,000	0.91	89.2	Negative	NA
14	COVID unit (general medicine)	NA	NA	Chest pain, cough, shortness of breath	9/30/20	10/4/20	No	10/4/20	10/5/20	**40**	**4,000**	**0.30**	260.1	**Positive**	33.04
15	COVID unit (general medicine)	NA	NA	Fever, shortness of breath, cough	10/2/20	10/4/20	No	10/4/20	10/5/20	**40**	**4,000**	**0.30**	260.1	**Positive**	36.27
16	Household bedroom	Yes	No	Respiratory symptoms, fatigue	NA	NA	No	10/5/20	10/7/20	**40**	**2,000**	**0.91**	156.1	**Positive**	36.1
16	Household bedroom	Yes	No	Respiratory symptoms, fatigue	NA	NA	No	10/5/20	10/7/20	**40**	**2,000**	**1.83**	156.1	**Positive**	37.43
17	Household bedroom	No	No	Respiratory symptoms, fatigue	NA	NA	No	10/5/20	10/8/20	20	1,000	0.91	136.6	Negative	NA
17	Household tv room	Yes	No	Respiratory symptoms, fatigue	NA	NA	No	10/5/20	10/8/20	20	1,000	0.91	53.5	Negative	NA
18	Household kitchen	No	No	Respiratory, loss of taste/smell	NA	NA	No	10/1/20	10/8/20	**20**	**1,000**	**0.91**	53.5	**Positive**	37.65
18	Household living room	No	No	Respiratory, loss of taste/smell	NA	NA	No	10/1/20	10/8/20	**20**	**1,000**	**NA**^a^	66.9	**Positive**	37.52
19	Household bedroom	Yes	No	Respiratory symptoms	NA	NA	No	10/6/20	10/9/20	20	1,000	NA^a^	47.6	Negative	NA
19	Household tv room	Yes	No	Respiratory symptoms	NA	NA	No	10/6/20	10/9/20	20	1,000	0.91	74.3	Negative	NA
20	Household bedroom	No	Yes	Respiratory symptoms, fatigue	NA	NA	No	8/3/20	8/6/20	**20**	**1,000**	**1.83**	89.2	**Positive**	37

Note. ICU, intensive care unit; NA, not applicable; RT-PCR, reverse-transcriptase polymerase chain reaction; CT, cycle threshold; BiPAP, bilevel positive airway pressure.

a No positive patient was present in the room.

### Hospital air samples

Of 15 patient rooms sampled, 14 were located in COVID-19 units (5 in ICUs and 9 in general medicine wards) and 1 was located on a non–COVID-19 unit. All rooms were set to have at least 6 air changes per hour, with negative pressure relative to the hallway, and their median size was 260.1 m^2^ (range, 89.2–260.1). Of 15 air samples, 7 sampled 1,000 L (range, 1.83–0.91 m from the patient), and all of these samples were SARS-CoV-2 negative. Moreover, 5 samples tested 2,000 L at 0.91 m from the patient (all SARS-CoV-2 negative), 2 samples tested 4,000 L at 0.91 m (all SARS-CoV-2 negative), and 2 patients were sampled using 4,000 L each at 0.30 m from the patient (both SARS-CoV-2 positive) (Table 1 and Supplementary Fig. 1 online).^9^

Regarding the characteristics of the 15 inpatients housed within the rooms sampled: 5 (33%) had hypoxic respiratory failure, 4 (26.6%) had COVID-19 pneumonia, 3 (20%) had fever with respiratory symptoms, 2 (13.3%) were asymptomatic, and 1 (6.6%) had septic shock of unclear etiology (Table 1). Two-thirds of patients required oxygen support at the time of sampling: 2 (13.3%) were mechanically intubated, 2 (13.3%) were on bilevel positive airway pressure, 3 (20%) were on high-flow nasal cannulae, and 4 (26.6%) were on nasal cannulae. The median number of days from symptom onset to air sampling was 5 (range, 2.75–8.5). The median number of days from the last positive SARS-CoV-2 test to the day of air sampling was 1.5 (range, 1–3.75). The 2 patients occupying the rooms with positive air samples had mild severity of illness and were not on supplemental oxygen. Days from symptom onset to sampling were 5 (patient 14) and 3 (patient 15).

### Household air samples

All 5 households had at least 1 symptomatic SARS-CoV-2–positive member at the time of sampling, and nonoe of these patients was on supplemental oxygen. All positive household members had respiratory symptoms at the time of air sampling. The median number of days from the last positive SARS-CoV-2 test to the day of air sampling was 3 (range, 2.5–5). Furthermore, 5 samples (55.5%) from 3 households were positive for SARS-CoV-2 (Supplementary Fig. 2 online). Only 1 household had air conditioning running (no. 20), and 3 households had opened windows or doors immediately prior to air sampling (nos. 16, 17, and 19). Anecdotally, most households felt warm and humid at the time of testing.

## Discussion

In this study, household samples were 8 times more likely to test positive for SARS-CoV-2 than inpatient samples. Inpatient rooms only tested positive when the volume of air sampled was quadrupled and the distance between air samplers and patients was minimal. Thus, these positive results may represent contaminated respiratory droplets being expelled by patients rather than actual air contamination. Given that room ventilation (ie, air changes per hour) was the main difference between these settings, our findings may suggest that the degree of ventilation in a room is more important in determining the degree of air contamination than the acuity of illness that a SARS-CoV-2 patient may be experiencing. Previous studies have characterized the air contamination in inpatient areas with a wide range of findings between 1.3% and 63.2%^2-5^; however, the viability of the virus in air samples is still controversial. To the best of our knowledge, this is the first report of air contamination by SARS-CoV-2 within household settings.

Our study has several limitations. The sample size was small; we used convenience samples and did not perform viral cultures. Furthermore, we did not measure the temperature or humidity of the rooms, which are environmental variables that may potentially affect the viability of the virus. In addition, we obtained more air samples per household than per inpatient room; therefore, we increased the likelihood of detecting positive samples in households. Despite these limitations, our preliminary findings suggest that household settings may have high degree of air contamination, signaling a major impact of room ventilation on this outcome. Future studies should characterize the variables determining the degree of air contamination in households and explore innovative ways to ameliorate this problem, especially in crowded households without access to natural Please provide missing Acknowledgments text?ventilation.

## Supplementary material

For supplementary material accompanying this paper visit http://dx.doi.org/10.1017/ice.2021.45. click here to view supplementary material
