# Supplementary material for: Air contamination of households versus hospital inpatient rooms occupied by severe acute respiratory coronavirus virus 2 (SARS-CoV-2)–positive patients
Source: Infect Control Hosp Epidemiol. 2021 Feb 4:1–5. doi: 10.1017/ice.2021.45 (PMC8047394; doi:10.1017/ice.2021.45)
Supplement: Supplementary file 1 [file icesup.zip › S0899823X21000453sup001.docx]

Supplementary Figure 1.

Title: Layout of hospital rooms designated for COVID-19 patients and position of air samplers.

Legend: The air sampler was placed on the side table at A) 6 feet and B) 3 feet from the patient. C) Two air samplers were run at one foot from the patient’s face. Red circle: air sampler; Red cross: patient’s face. Horizontal surfaces are depicted in blue.

Supplementary Figure 2.

Title: Household settings positive for SARS-CoV-2.

Legend: A) Household 16: the two positive samples were collected from the same room at 3 and 6 feet from the patient; B) Household 18 had positive samples in the living room and the kitchen; symptomatic children were running around the household at the time of collections (dotted line), but no one was in close proximity to the air sampler placed in the living room. The kitchen air sampler ran while one of the symptomatic adults cooked. C) Household 20: the air sample was obtained while the symptomatic patient talked on her cellphone during sampling. Red circle: air sampler; Red cross: patient’s face. Horizontal surfaces are depicted in blue.
