# Supplementary figures and images for: Air contamination of households versus hospital inpatient rooms occupied by severe acute respiratory coronavirus virus 2 (SARS-CoV-2)–positive patients
Source: Infect Control Hosp Epidemiol. 2021 Feb 4:1–5. doi: 10.1017/ice.2021.45 (PMC8047394; doi:10.1017/ice.2021.45)

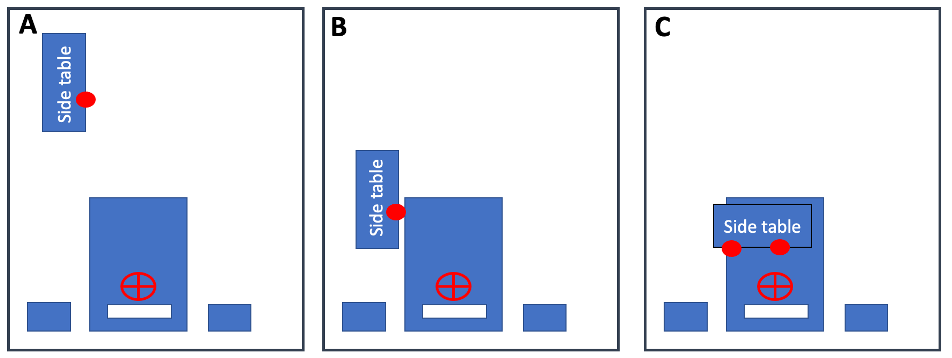

Supplement: Supplementary file 1 [file icesup.zip › S0899823X21000453sup002.png]

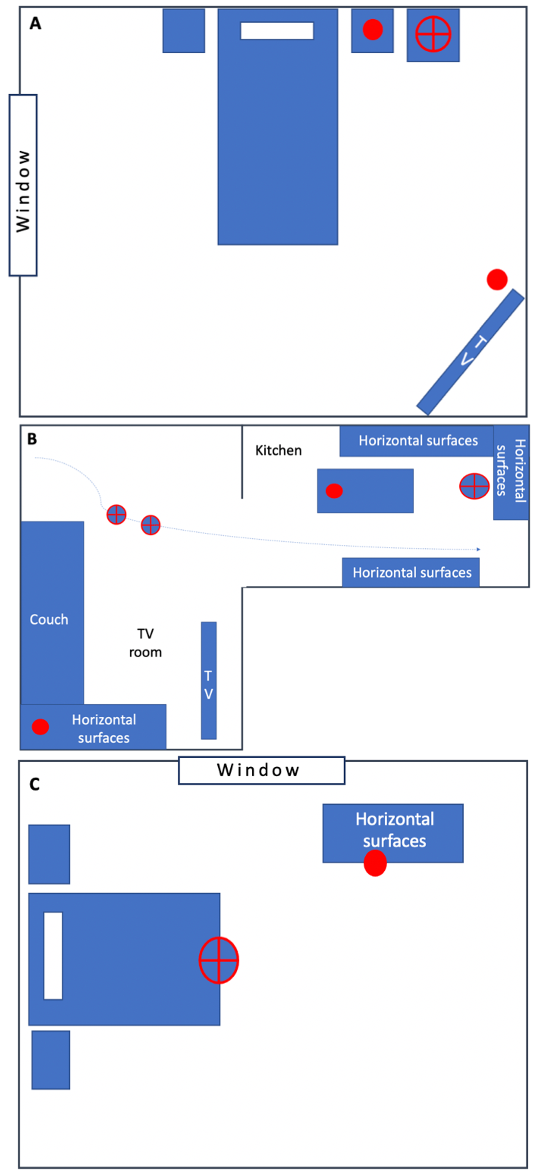

Supplement: Supplementary file 1 [file icesup.zip › S0899823X21000453sup003.png]
